# SOCIAL RELATIONSHIPS OF OLDER ADULTS DURING THE COVID-19 PANDEMIC

**DOI:** 10.1093/geroni/igae098.2545

**Published:** 2024-12-31

**Authors:** Tina Kilaberia, Yuanyuan Hu, Janice Bell

**Affiliations:** New York University, New York, New York, United States; New York University, New York, New York, United States; UC Davis, Davis, California, United States

## Abstract

This study examined the impact of the COVID-19 pandemic on social relationships of older adults in two groups: eleven who were part of a caregiving dyad and seven who were not (mean age=79; 12 White, 5 Black, 1 More Than One Race). Semi-structured qualitative interviews were combined with the Lubben Social Network Scale (six-item version, LSNS-6). Those in dyads and not in dyads reported comparable extent of social connections on average with family (9 monthly social engagements vs. 9.5 on LSNS-6, respectively). Those in dyads reported greater social engagement on average with non-family individuals than those not in dyads (8.5 vs. 7 on LSNS-6). Older adults in both groups had greater social connections with family than non-family members. Qualitative themes such as seeking, losing and keeping ties represent the range of social connection experiences: all in dyads (vs. five not in dyads) referred to family members as key persons during the pandemic, five (vs. one) reported social isolation from family members specifically, zero (vs. three) identified a spouse or partner as most protective, five (vs. three) described supportive relationships, and four (vs. one) described relationships needing adjustments as a result of the pandemic’s impact. Six older adults in dyads (vs. two not in dyads) described non-family contacts as key, eight (vs. three) described the loss of connection with friends, and three (vs. zero) identified friends or hobbies as most protective. Type of dyadic relationships (spouses vs. parent-child vs. friends) and relationship quality may influence older adults’ social connections differently.

